# Controlled EGCG Release from Zeolite-Coated Ti6Al4V: Effects of Divalent Ions on Release and Cytotoxicity

**DOI:** 10.1021/acsbiomaterials.5c01572

**Published:** 2026-01-21

**Authors:** Wiktoria Stachowicz, Alicja Wojcik, Dominika Podbereska, Maria Ratajczak, Adam Voelkel, Agata Przekora, Mariusz Sandomierski

**Affiliations:** † Institute of Chemical Technology and Engineering, Faculty of Chemical Technology, 49632 Poznan University of Technology, Berdychowo 4, 60-965 Poznan, Poland; ‡ Department of Tissue Engineering and Regenerative Medicine, 49554 Medical University of Lublin, Chodzki 1, 20-093 Lublin, Poland; § Institute of Mechanical Technology, 49632 Poznan University of Technology, ul. Piotrowo 3, 60-965 Poznań, Poland; ∥ Institute of Building Engineering, 49632 Poznan University of Technology, Piotrowo 5, 60-965 Poznan, Poland

**Keywords:** epigallocatechin gallate, titanium alloys, zeolites, osteoporosis, drug delivery

## Abstract

This study presents the development of titanium-based implants coated with zeolite layers for controlled delivery of epigallocatechin gallate (EGCG), a polyphenolic compound with osteogenic, antiresorptive, and antibacterial properties. Zeolite coatings were modified with divalent ions (Zn^2^^+^, Mg^2^^+^, Ca^2^^+^) to investigate their influence on EGCG adsorption and release under neutral (pH 7.4, SBF) and acidic (pH 5.0, acetate buffer) conditions. Comprehensive characterization using SEM, EDS, FT-IR, UV–vis spectroscopy, and surface profilometry confirmed uniform zeolite formation, effective EGCG loading, and tunable release profiles. Zinc-containing zeolite exhibited the highest EGCG adsorption but demonstrated cytotoxicity toward hFOB 1.19 osteoblasts. Magnesium-zeolite-coated implants provided controlled EGCG release, were nontoxic, and did not support cell adhesion, making them suitable for temporary internal fixation in the management of orthopedic trauma. Release studies revealed pH-dependent kinetics, with accelerated EGCG release under acidic conditions simulating osteoclast activity. These findings demonstrate the potential of Mg-zeolite-coated titanium implants as functional devices that provide mechanical support, enable localized drug delivery, and promote bone regeneration while minimizing tissue damage during removal.

## Graphical abstract

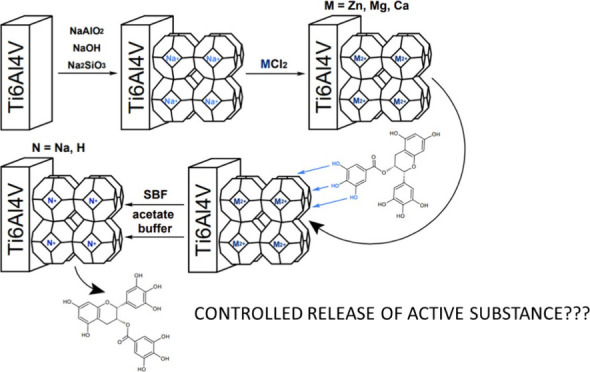

## Introduction

Biomaterials are a group of materials with specific structures and properties, the most important being their biocompatibility. They have found numerous applications in implantology, enhancing patients’ quality of life and restoring the function of damaged tissues and organs.^1^ The utilization of biomaterials for implants necessitates the consideration of numerous variables including biotolerance, corrosion resistance, and mechanical properties to ensure their safe deployment.^2^ Biotolerance involves the absence of toxic, sensitizing, immunogenic, or carcinogenic reactions, along with chemical stability and inertness.^1^ High corrosion resistance is crucial because corrosion products may cause undesirable physiological reactions in the body and lead to implant rejection.^3^ A pathological condition, which may arise when an implant is subjected to excessive loads, can lead to a dominance of bone resorption over the formation of new bone matrix. Mechanical incompatibility can lead to a decrease in bone density, thus reducing mechanical strength and increasing the risk of low-energy fractures.^4^

Metallic biomaterials, mainly titanium and its alloys, have attracted considerable interest in recent years for biomedical applications. This interest is largely due to the highly favorable properties and the ability of titanium implants to remain functional in the body for over 20 years.^5^ Titanium medical devices provide long-term structural and mechanical support during the reconstruction of hard tissue or the replacement of its function.^6^ Research has shown that titanium exhibits high chemical and corrosion resistance due to self-passivation, which forms an oxide or hydroxide protective layer.^7^ For this reason, they provide an effective carrier material and matrix for bone-integrated medical devices.

The topography and composition of the implant surface are crucial for achieving adequate biocompatibility.^8^ The development of improved bioactive coatings, mainly ceramics, is a key objective to enhance osteoconduction, increase implant strength, and improve patient outcomes following surgery.^9^ The bioceramic layer must possess an appropriate porosity and pore size. Pores facilitate the transport of oxygen, nutrients, and metabolic products, which is crucial for local bone remodeling at the implantation site.^10^ A ceramic-metal composite allows the fabrication of implants with tailored and enhanced properties. If necessary, the material can remain in the body for extended periods without adverse reactions or requiring reimplantation.^11^

Zeolites are noteworthy materials among ceramic biomaterials. They represent aluminosilicates in crystalline form.^12^ They possess unique properties, including high biocompatibility, large specific surface area, and tunable pore size and physicochemical properties via modification of the silicon-to-aluminum ratio.^13^ A low silicon-to-aluminum ratio enhances the ion-exchange properties of zeolites, which is crucial for designing controlled drug delivery systems.^14^ Ion exchange in biological fluids allows for the safe release of drug molecules at targeted sites within the body.^15,16^

The above-mentioned solution represents an optimal approach for supporting individuals with osteoporosis. This condition is associated with alterations in the bone microstructure that result in reduced strength, thereby increasing the risk of low-energy fractures.^17^ The symptoms of progressive disease typically manifest at an advanced stage, when fractures occur and pharmacological interventions are no longer effective. At this stage, it may be necessary to reinforce the fractured bone with a temporary implant, such as a screw, a plate, or other fixation device, or to replace the weakened bone with a permanent implant.^18^ In the case of temporary implants (internal orthopedic fixation) used to stabilize a fractured bone, they should be easily removable after the bone has healed; therefore, bone overgrowth on their surface should be avoided. In contrast, for permanent bone replacement implants, bone ingrowth is essential. In both cases, there is an ongoing need to develop implants with enhanced properties compared to those currently available.

One of the most recent advancements in the treatment of bone diseases, such as osteoporosis, is the development of implants that function as drug delivery systems. These implants not only replace compromised bone but also temporarily reinforce fractured bone during healing and help counteract the osteoporotic condition in the surrounding tissue. Unlike conventional drug delivery techniques for osteoporosis, implant-based release allows targeted action at specific sites of pathology.^14^ This study focuses on epigallocatechin gallate (EGCG), a flavonoid compound, as the active substance. EGCG is a principal polyphenolic constituent of green tea and is renowned for its multiple beneficial effects in the treatment of various conditions, including skeletal disorders.^19^ EGCG has been shown to possess antibacterial, antioxidant, and anti-inflammatory properties and to positively influence new bone formation.^20^ This effect is achieved by promoting the osteogenic differentiation of mesenchymal stem cells and inhibiting osteoclasts, the cells responsible for bone resorption.^21^ The behavior of EGCG largely depends on the surrounding pH, with evidence indicating higher stability at lower pH values.^22^ Osteoclasts, through the secretion of enzymes (including proteases) and hydrochloric acid, dissolve the inorganic bone matrix, thereby lowering the pH in the vicinity of the bone tissue.^23^ By combining the stability of EGCG in an acidic environment with its inhibitory effect on osteoclasts, it is possible to rapidly reduce the activity of bone-resorbing cells.

In this study, we aimed to develop modern titanium-based internal fixation capable of supporting the recovery of individuals who have suffered bone fractures. These implants are intended to function as temporary devices, similar to conventional devices such as plates, screws, rods, and wires that stabilize broken bones during healing, while also providing controlled delivery of therapeutic agents. Our approach involves coating the implants with zeolite layers and enabling the controlled release of EGCG from their surfaces. The research process is presented graphically in Figure 1. The topic of controlled drug delivery from implants is highly promising but remains underexplored. Delivering EGCG from such implants represents a novel strategy. Moreover, this study investigates the effect of divalent ions on prolonged EGCG release, as well as the influence of the surrounding pH on the release kinetics. The obtained materials were extensively characterized, confirming the successful formation of the zeolite layers, the loading of EGCG, and its controlled release. EGCG was shown to be evenly distributed across the entire surface of the implants. Divalent ions were found to influence both the release profile and the cytotoxicity of the materials. One of the developed implants demonstrated significant potential as a temporary fixation that can be safely removed without causing damage to the bone. Overall, the results demonstrate the feasibility of creating functional implants with controlled EGCG delivery and tailored properties for supporting bone regeneration, providing both mechanical support and localized therapeutic action during healing.

**Figure 1:**
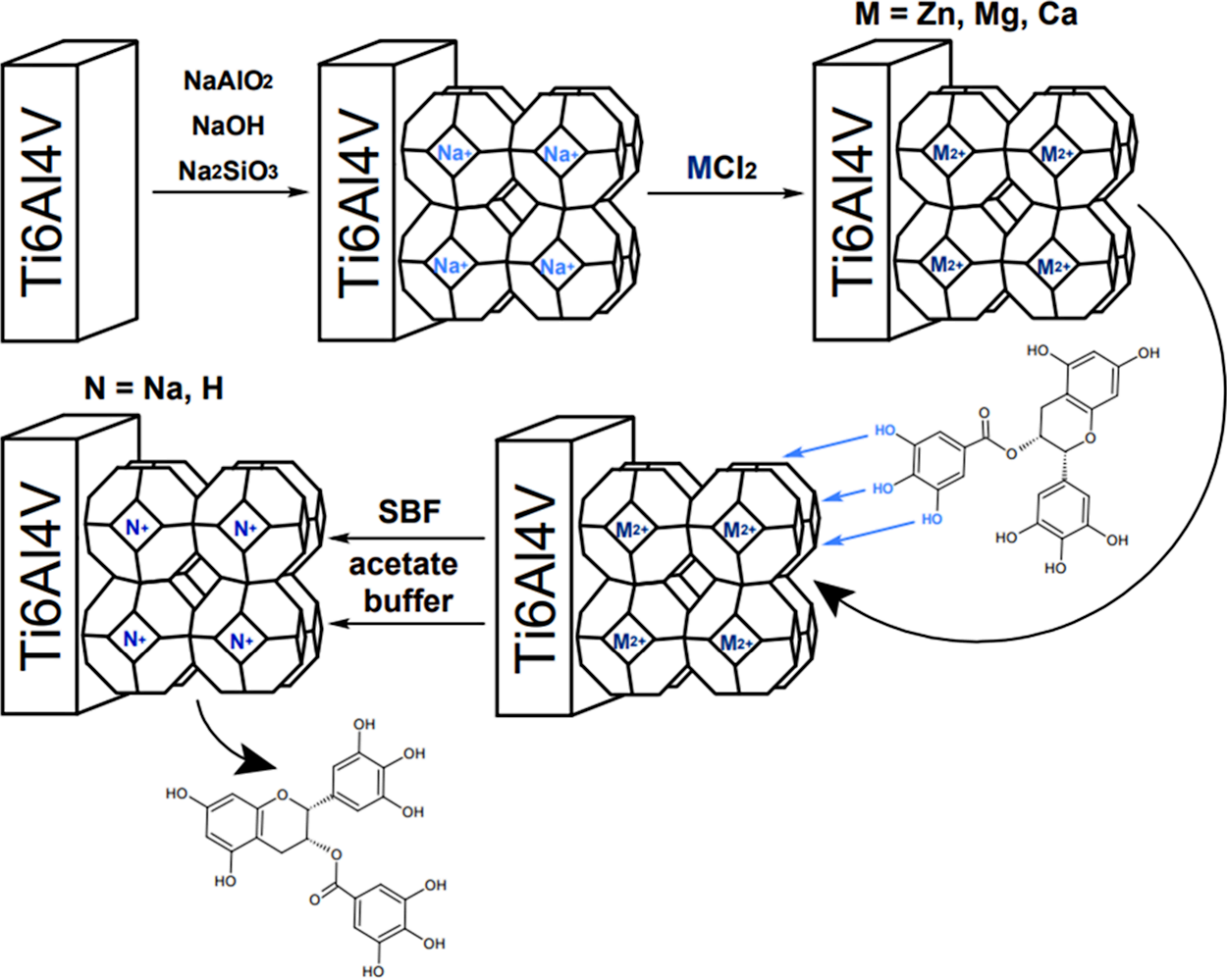
Graphical representation of the deposition of the zeolite layer, the sorption process, and release of the active substance.

## Materials and Methods

### Reagents

Sodium hydroxide, sodium silicate, epigallocatechin gallate (EGCG), potassium hydrogen phosphate trihydrate, and tris­(hydroxymethyl)­aminomethane (TRIS) were obtained from Sigma-Aldrich. Ethanol, acetone, hydrogen peroxide, zinc chloride, magnesium chloride, and acetic acid were obtained from CHEMPUR. Sodium aluminate, calcium chloride, sodium chloride, sodium bicarbonate, potassium chloride, sodium sulfate, and hydrochloric acid were obtained from POCH.

### Zeolite Coating Containing Sodium Ions

Zeolite coatings were prepared following the methodology described in the literature.^15^ The Ti6Al4V titanium alloy samples (diameter 8 mm, height 4 mm) were subjected to abrasion using 150-grit sandpaper. Next, the samples were cleaned with demineralized water, ethanol and acetone. Subsequently, samples were immersed in a 30% hydrogen peroxide solution at room temperature for 3 h and dried. The in situ hydrothermal method was employed for the production of zeolite coatings. 2.46 g of sodium aluminate was dissolved in the previously prepared sodium hydroxide solution (45.22 g in 200 g of H_2_O), and the resulting mixture was slowly added to 7.22 g of sodium silicate. The mixture was then stirred at room temperature for 30 min. The deposition of the zeolite coating was conducted at 80 °C for 5 h. After the time had elapsed, the samples were rinsed with demineralized water for 24 h. The process was repeated with a fresh portion of water until a neutral pH was achieved. The Ti-Zeo-Na material was obtained.

### Zeolite Coating Containing Zinc/Magnesium/Calcium Ions

The resulting material was placed in 20 mL of 0.5 M chloride solutions for 24 h, and the process was repeated three times for each type of ion: ZnCl_2_ – zinc zeolite, MgCl_2_ – magnesium zeolite, and CaCl_2_ – calcium zeolite. During this process, ion exchange occurred, whereby sodium ions were replaced by zinc, magnesium, and calcium ions. Subsequently, the material was washed three times with demineralized water and allowed to dry under cover. The following materials were obtained: Ti-Zeo-Zn, Ti-Zeo-Mg, Ti-Zeo-Ca.

### Sorption of the Active Substance

In order to facilitate the sorption of the active substance, EGCG, the modified samples were placed in Falcon tubes and each was poured with 5 mL of the prepared solution (0.1 mg EGCG per 1 mL H_2_O). The test tubes were subjected to agitation at room temperature for a period of 5 days. The change in the concentration of the active substance in the solution was analyzed at appropriate time intervals (1, 2, 3, 5 days) using the UV–vis method. The following materials were obtained: Ti-Zeo-Zn-EGCG, Ti-Zeo-Mg-EGCG, Ti-Zeo-Ca-EGCG. During the analysis of the EGCG sorption capacity, three independent replicates were performed for each material.

### Release of the Active Substance

Two environments with varying pH values were utilized to facilitate the release of the active substance from the surface of the modified titanium alloy. Two distinct solutions were employed: a simulated body fluid (SBF) with a high concentration of Na^+^ ions and a pH of 7.4, and an acetate buffer with a pH of 5.0. Samples containing individual ions (Zn^2+,^ Mg^2+^, Ca^2+^) were divided into two groups. The first half of the samples was each flooded with 1 mL of SBF, while each sample from the second half was flooded with 1 mL of acetate buffer.

The drug release was conducted over a seven-day period at a controlled temperature of 36 °C. The variation in the concentration of the active substance solution was evaluated through the use of UV–vis spectroscopy at the following time points:

- for SBF: 1 h, 2 h, 3 h, 1 day, 2 days, 7 days,
- for acetate buffer: 15 min, 30 min, 45 min, 60 min, 75 min, 90 min, 150 min, 1 day, 2 days, 7 days.

During the release analysis, three independent replicates were performed for each material in both SBF and acetate buffer.

Preparation of SBF - the following were dissolved in demineralized water: NaCl (8.035 g), NaHCO_3_ (0.355 g), KCl (0.255 g), K_2_HPO_4_·3H_2_O (0.231 g), Na_2_SO_4_ (0.072 g), TRIS (0.612 g), HCl (0–5 mL). TRIS was used as a buffering agent, and then the pH was adjusted to approximately 7.4 by titration with 1 M HCl.

The acetate buffer was prepared by combining solutions A and B in the appropriate proportions.

A: 115 mL of glacial acetic acid was measured and added to 100 mL of H_2_O,

B: 72 g of sodium acetate was weighed and mixed with 100 mL of H_2_O.

Subsequently, 37 mL of Solution A and 88 mL of Solution B were transferred to a separate vessel and replenished with distilled water to a volume of 250 mL. The entire mixture was then stirred for 1 h. Next, the pH was adjusted to 5.0 using a 5% acetic acid solution.

The objective of using two distinct pH environments was to examine the EGCG release profile in relation to the acid–base balancing mechanisms within the body. Given the sensitivity of cell remodeling mechanisms to changes in the concentration of H^+^ ions in the environment, precise maintenance of the pH of the blood and extracellular fluid is of significant importance. Numerous studies have shown that osteoclasts, cells that are responsible for the resorption of bone tissue, exhibit minimal activity at a pH of 7.4. However, their high sensitivity to pH changes means that a decrease of just 0.1 can result in their activation. This, in turn, triggers unfavorable processes, including the secretion of enzymes that resorb bone tissue, further lowering the local pH. We selected an acetate buffer at pH 5.0 to simulate the acidic microenvironment created by osteoclasts during bone resorption (typically pH ∼ 4.5–5.5).^24–27^

### Characterization of Materials

#### Scanning Electron Microscopy (SEM) and Energy Dispersive Spectroscopy (EDS)

Scanning electron microscopy (SEM) images were obtained using a VEGA device (TESCAN, Czech Republic), which was also equipped with an energy-dispersive spectroscopy (EDS) analyzer (Bruker, Germany). The device generates an image of the analyzed sample by scanning the surface with a focused electron beam. The interaction of electrons with the atoms that comprise the sample generates signals that contain information regarding the surface topography and the composition of the sample.

#### Surface Topography Measurements

Surface topography measurements were performed using a Bruker Alicona IF PortableRL optical profilometer. A scanned sample area of 2 × 2 mm was scanned at 20× magnification. The surface topography measurements were performed according to ISO standard 25178. The areal surface texture parameters (S-parameters) were taken with a cutoff wavelength (λc) of 250 μm.

The coating thickness was measured as follows. For coatings where the base material was not visible, a small section of the coating was carefully removed to expose the raw material. This step made it possible to measure the coating’s thickness. The thickness was then measured using the Alicona MeasureSuite 5.3.2 software. A total of five measurements were taken, and the mean and standard deviation were calculated from these values.

#### Fourier Transform Infrared Spectroscopy (FT-IR)

The distribution of zeolite and active substances was analyzed using an FT-IR LUMOS II microscope (Bruker, Germany). Imaging was conducted over an area of 1000 × 1000 μm. The location of EGCG was determined based on the analysis of the area of peaks occurring in the range of 1450–1300 cm^–1^. A total of 60 scans were carried out for each spectrum. The results were obtained in ATR mode. The data were processed using OPUS 8 software (Bruker).

#### UV–Vis Spectroscopy

The changes in the concentration of active substance during their adsorption on the surfaces of the modified titanium alloy under the influence of SBF and acetate buffer were determined using a UV–vis–UV-2600 spectrophotometer (Shimadzu, Japan). Measurements were conducted for both the sorption and release processes within the wavelength range of 400–250 nm, with a maximum at 273 nm. In the sorption process, water was employed as a background, while in the release process, SBF and acetate buffer were utilized. The retained amount of the active substance was calculated based on the curve obtained for EGCG in water, while the released amount was calculated using the EGCG curve in SBF and acetate buffer, respectively.

### Biological Evaluation

#### Cells

A commercially available normal human fetal osteoblast cell line (hFOB 1.19 cell line, CRL-3602; purchased from ATCC-LGC Standards, Teddington, UK) was employed, as it is considered an excellent cellular model due to its close resemblance to primary osteoblasts. The cells were maintained in the DMEM/Ham’s F12 medium without phenol red (Sigma-Aldrich Chemicals, Warsaw, Poland), supplemented with 10% fetal bovine serum (Pan-Biotech GmbH, Aidenbach, Bavaria, Germany), 100 U/mL penicillin, 100 μg/mL streptomycin, and 300 μg/mL G418 (Sigma-Aldrich Chemicals, Poland). The cells were incubated at 34 °C in a humidified atmosphere with 5% CO_2_.

#### Cytotoxicity Assessment

The cytotoxicity of the fabricated materials was evaluated quantitatively and qualitatively. The experiments were conducted in accordance with the ISO 10993-5 standard for the biological evaluation of medical devices (2009). The cells were cultured in 96-well plates at a density of 2 × 10^4^ cells per well in 100 μL of growth medium and allowed to adhere for 24 h to reach the appropriate confluency. Following incubation, the medium was replaced with material extracts. The extracts were prepared in accordance with ISO 10993-12 by incubating the test samples in complete culture medium at a surface area-to-volume ratio of 1.25 cm^2^/mL at 37 °C in a humidified atmosphere with 5% CO_2_ for 24 h. As a negative control for cytotoxicity, culture medium incubated in a polystyrene tube was used. After 24 h of exposure to the extracts, cell viability was evaluated using the MTT assay (Sigma-Aldrich Chemicals, Poland). Results were expressed as a percentage of the absorbance measured for the negative control group, and the material was considered noncytotoxic if cell viability exceeded 70%.

Cytotoxicity test was performed in sextuplicate in three independent experiments. The results obtained with MTT assay were checked for statistically significant differences (*p* < 0.05) among tested groups (all groups were compared to each other, including control cells) using one-way ANOVA test followed by Tukey’s multiple comparison test (GraphPad Prism 5, Version 5.03)

Qualitative cytotoxicity assessment was performed using live/dead staining of cells cultured on the implant surface. Before the experiment, the biomaterials were preincubated for 1 h in 400 μL of the complete growth medium suitable for hFOB 1.19 cell line. Then, hFOB 1.19 cells were seeded (density 2 × 10^5^/mL) on the biomaterials in 600 μL of growth medium and allowed to grow for 4 days, 34 °C, 5% CO_2_, 95% of air humidity with medium renewal every 2 days. After 4 days of culture, the implants were subjected to viability staining using calcein-AM (green fluorescence indicating live cells) and propidium iodide (red fluorescence marking nuclei of dead cells), both components of the live/dead staining kit (Sigma-Aldrich Chemicals, Poland). The staining was carried out following the manufacturer’s protocol. Then, the cells were visualized by confocal laser scanning microscope (CLSM, Olympus Fluoview equipped with FV1000).

#### Cell Proliferation Assessment

Because produced samples do not support cell adhesion, proliferation assay was performed indirectly using cell culture inserts. The materials were placed into cell culture inserts with pore diameter 3 μm and incubated with the hFOB 1.19 cells that were cultured on the bottom of polystyrene well.

After 1, 2, and 3 days of culture, the number of cells was determined by using colorimetric WST-8 test (Sigma-Aldrich Chemicals, Poland) which was performed according to the manufacturer protocol. The cell number was estimated from the calibration curve, made for known concentrations of hFOB 1.19 cells.

The results obtained with WST-8 assay were checked for statistically significant differences (*p* < 0.05) among tested groups (all groups were compared to each other, including control cells) using one-way ANOVA test followed by Tukey’s multiple comparison test (GraphPad Prism 5, Version 5.03).

## Results

### Material Characterization

The first part of the study focused on evaluating the efficiency of zeolite layer formation on the titanium surface, performing a comprehensive characterization of the resulting coatings, and determining the effect of ions on EGCG adsorption and release.

The SEM analysis, which was conducted on all samples (Figure 2), confirmed the formation of microporous surfaces, thus confirming the successful modification of the titanium alloy. It is evident that the zeolite coating is distributed uniformly across the titanium material. No significant changes are observed in the 5000× scans before and after adsorption of the active substance. This suggests that EGCG was attached to the surface as a result of ion exchange, rather than precipitation. The images obtained before and after the sorption process demonstrate the presence of discrete zeolite crystals with a diameter of 2–3 μm. Based on the morphology, it can be concluded that sodalite was obtained.^28^

**Figure 2:**
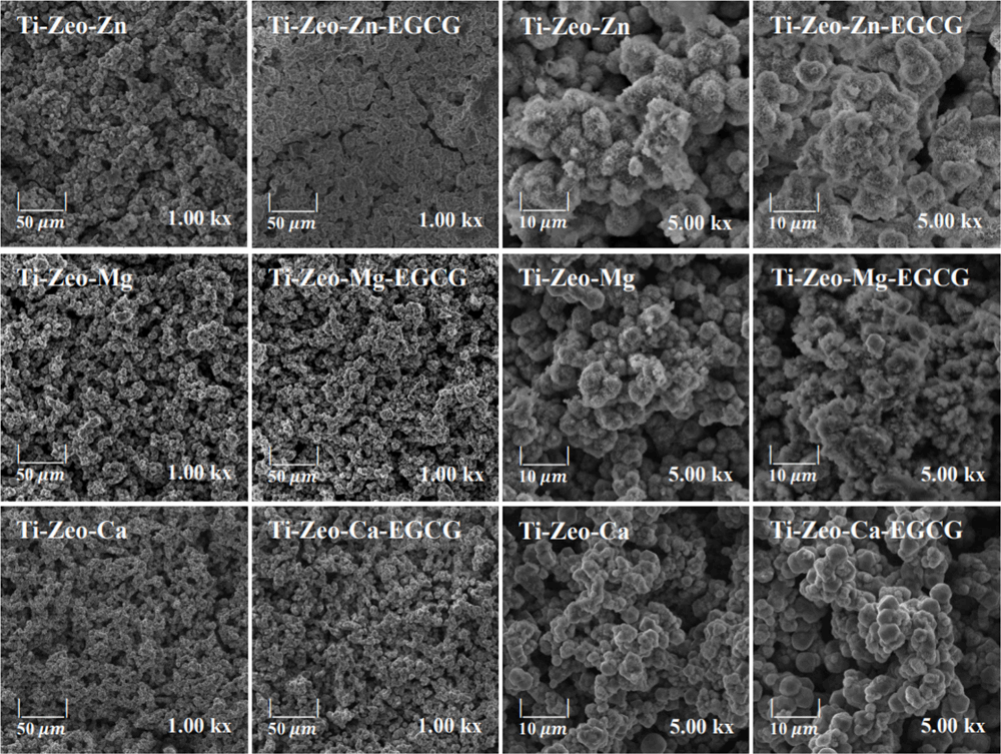
SEM photos of surfaces modified with zinc, magnesium, and calcium zeolite before and after the EGCG sorption process. The images show the magnification used and the scale in order to present the grain size.

At 1000× magnification, the images of the samples containing zinc zeolite after the addition of EGCG show a flatter, more compact structure. This may be attributed to the substantial attachment of the active substance to their surface. However, due to the least noticeable differences between the photos for calcium zeolite, it can be concluded that the sorption process was not effective for this material.

In the next stage of the study, the surface topography of the prepared materials was determined (Table 1). The surface characteristics of an implant play a critical role in the bone healing process and bone-implant interface. Surface roughness, at both micro- and macrostructural levels, significantly influences the biomechanical properties of the implant by enhancing mechanical retention (interdigitation) and providing good stress distribution. Surface modifications - achieved through mechanical or chemical treatments (for example, coating techniques) - aim to improve cell adhesion, biomineralisation, and bonding between the implant and bone by increasing the effective contact area. An increase in implant surface roughness not only enlarges the interfacial area with bone tissue but also enhances biomechanical interlocking and improves osseous stability. Researchers have shown that higher average surface roughness promotes cellular responses conducive to bone formation.^29–32^ The surface modifications used in this study change the material’s topography. This process creates a rough surface (Sa > 2 μm) with highly developed structure (Sdr > 50%), which increases the actual area available for biological interactions compared to the original material.^33^ The modification with the EGCG coating causes an additional increase in both roughness and complexity, which may means a larger biologically active surface area. The surface topography was presented using microscopic imaging (Figure 3). The image is shown as a height map, where the lowest areas are marked with violet and the highest with red. This representation confirms that a complex structure was formed. A qualitative analysis of these areas shows that there is a majority of either valleys (colors from blue to violet) or peaks (areas from light green to red, rising above the average plane). However, it should be noted that uneven coating distribution may cause parts of the base material to remain exposed (visible as dark purple areas), which can influence the average values of topographical parameters measured over the whole analyzed surface. Although a properly rough implant surface brings clear benefits, it is important to consider the potential risks of making the surface too rough. Highly rough surfaces, especially those with many deep valleys (positive profile skewness, Ssk > 0), can create ideal conditions for bacterial growth and biofilm formation. The presence of biofilm significantly increases the risk of peri-implantitis — an inflammation of the tissue around the implant, which is a serious complication that can lead to the loss of the implant.^29,34^ Therefore, implant surface design requires a careful balance between promoting osseointegration and minimizing the risk of complications. The ideal implant should improve biocompatibility and support osteogenesis, while also having antimicrobial properties. At the same time, it must provide the necessary mechanical support and limit bacterial adhesion.

**Figure 3:**
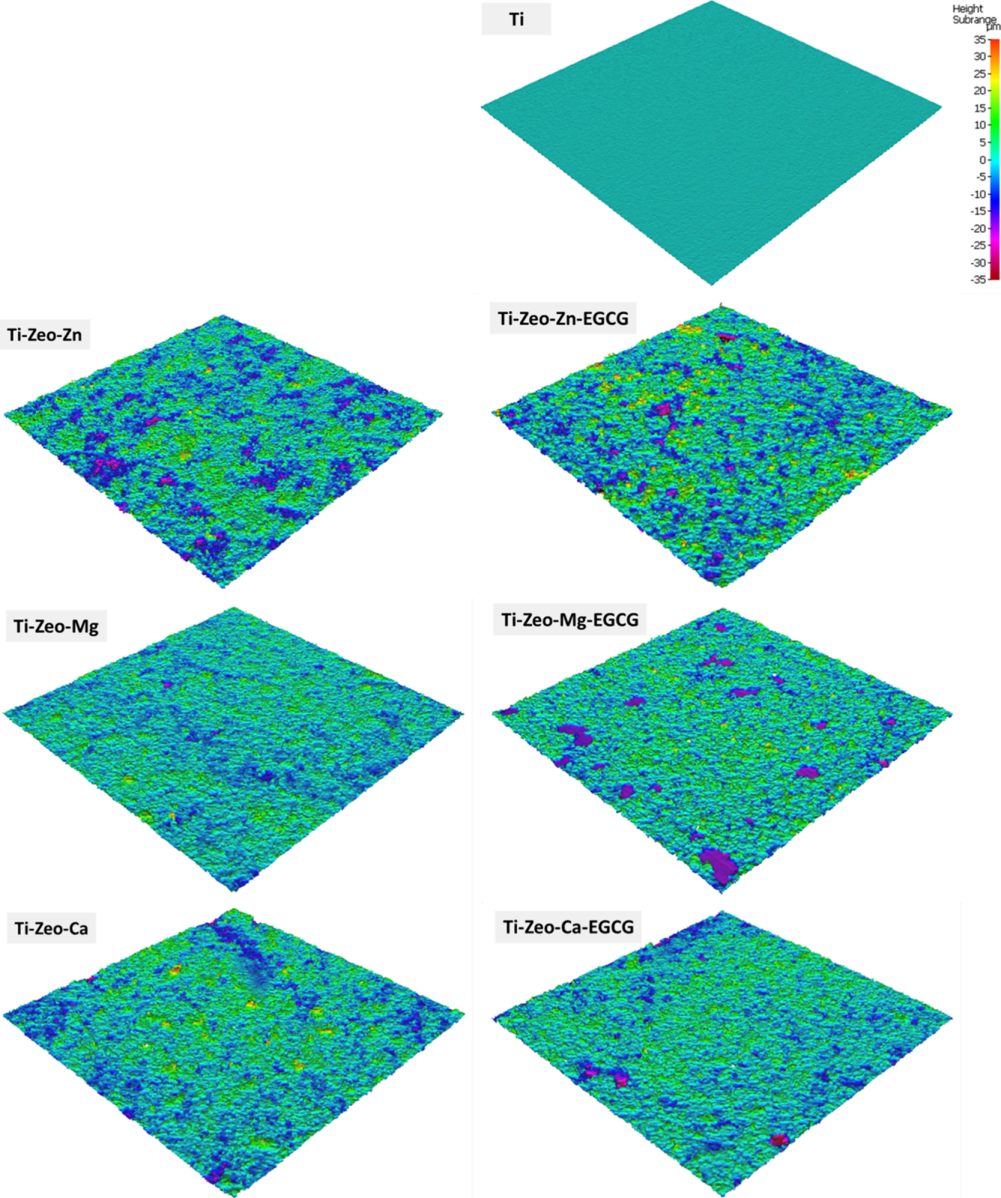
Surface topography of the sample measured using an optical profilometer. A 2 × 2 mm area was scanned at a 20× magnification.

**Table 1:** Parameters Determined Using the Optical Profilometer^a^

	Sa [μm]	Sq [μm]	Ssk	Sdr [%]	layer thickness [μm]
**Ti**	0.125	0.159	0.114	0.140	
**Ti-Zeo-Zn**	3.980	5.117	–0.028	70.736	27.63 ± 4.90
**Ti-Zeo-Mg**	2.867	3.749	0.259	31.618	25.87 ± 3.30
**Ti-Zeo-Ca**	3.686	4.832	0.121	54.484	20.96 ± 5.52
**Ti-Zeo-Zn-EGCG**	5.157	6.696	0.027	87.548	23.49 ± 4.63
**Ti-Zeo-Mg-EGCG**	4.444	5.682	–0.189	107.766	22.49 ± 4.42
**Ti-Zeo-Ca-EGCG**	3.832	4.919	–0.261	68.877	24.41 ± 3.59

a Sa: Arithmetical mean surface roughness. Sq: Squared mean surface roughness. Ssk: Skewness of the selected area. Sdr: Developed interfacial area ratio.

The EDS system’s mapping functionality enabled the analysis of dislocations at the individual element level on the surfaces of the tested samples. The images obtained for the materials after ion exchange show the homogeneous distribution of zinc, magnesium, and calcium ions across the entire zeolite surface (Figure 4), which may indicate the efficacy and efficiency of the ion exchange process. Comparative analysis of the SEM images and EDS mapping data reveals the presence of unstained regions, attributed to the formation of complex zeolite crystals, which contain free spaces between them.

**Figure 4:**
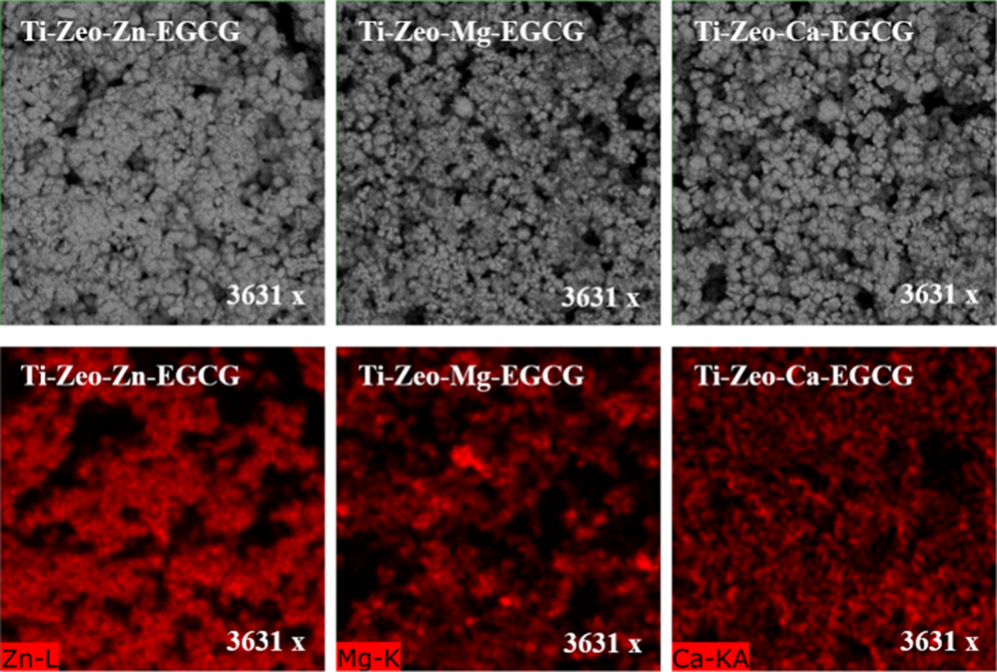
SEM images with EDS elemental maps showing the distribution of zinc, magnesium, and calcium ions on titanium surfaces modified with a zeolite layer after the EGCG sorption process. The SEM images indicate the specific areas where the EDS maps were acquired.

The ratio of silicon to aluminum has a significant impact on the pore size of the resulting material, which in turn affects its properties and strength. According to the results presented in Table 2, the Si/Al ratio was determined for samples with individual divalent ions. The values were 0.65, 1.17, and 1.29, respectively, for zinc, magnesium, and calcium zeolite. The data indicate that a material with a low silicon content is obtained, exhibiting high hydrophilicity and strong interactions with polar molecules.^14^ The low Si/Al ratio has a beneficial impact on the ion-exchange properties of the modified zeolite materials. The result obtained for the zinc zeolite differs, which may be related to the attachment of a significant amount of zinc ions to the zeolite surface. This, in turn, reduces the detectability of the sample in its deeper layers. The detected zinc content is over three times higher than magnesium and over four times higher than that of calcium. The difference in carbon content on the samples after the sorption process compared to before may indicate the efficient attachment of EGCG to the surface of the modified materials. Due to its cyclic aromatic structure, EGCG contains a considerable number of carbon atoms. Table 2 shows that the greatest amount of EGCG was attached to the surface modified with zinc zeolite. An increase was also observed for magnesium, whereas no increase was seen for calcium, suggesting that EGCG was not retained. The structure of the resulting zeolite-sodalite explains the presence of chlorine in the analyzed samples, as it is an aluminosilicate with the formula Na_8_[Al_6_Si_6_O_24_]­Cl_2_.^35^

**Table 2:** Amount of Elements Detected Using the EDS System

	Ti-Zeo-Zn	Ti-Zeo-Zn-EGCG	Ti-Zeo-Mg	Ti-Zeo-Mg-EGCG	Ti-Zeo-Ca	Ti-Zeo-Ca-EGCG
at. % ± stand. dev.						
Zn	15.7 ± 3.3	15.8 ± 3.6				
Mg			4.6 ± 2.3	4.1 ± 1.7		
Ca					3.5 ± 0.6	3.2 ± 1.6
Al	10.5 ± 1.3	10.5 ± 1.4	12.0 ± 1.5	12.8 ± 2.8	11.6 ± 1.9	11.3 ± 1.1
Si	6.9 ± 1.5	6.0 ± 1.5	14.1 ± 1.7	15.8 ± 1.8	15.0 ± 2.0	14.3 ± 1.8
C	4.4 ± 1.6	7.6 ± 3.5	2.5 ± 0.5	4.5 ± 1.9	2.3 ± 0.2	2.1 ± 0.6
Cl	3.6 ± 0.5	2.7 ± 0.3	0.6 ± 0.1	0.0 ± 0.0	0.0 ± 0.0	0.0 ± 0.0

The presence of the relevant functional groups in the zeolite structure was confirmed by Fourier transform infrared spectroscopy (FT-IR). Moreover, the analysis validated the efficient sorption of EGCG on the modified samples (Figure 5).

**Figure 5:**
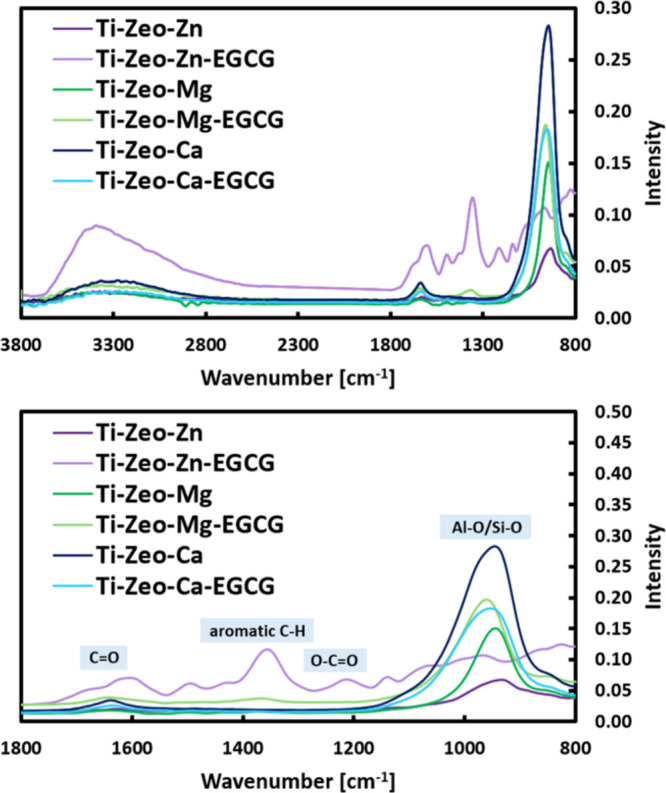
FT-IR spectra of titanium alloy surfaces modified with zinc, magnesium, and calcium zeolite (Ti-Zeo-Zn, Ti-Zeo-Mg, and Ti-Zeo-Ca) before and after EGCG sorption. The key bands observed in the samples are marked on the spectra.

The presence of a zeolite layer was confirmed by the observation of characteristic bands. The bands situated at 3300 and 1640 cm^–1^ arise from the stretching and bending vibrations of water that has been adsorbed on the surface, as well as from hydroxyl groups.^36,37^ The characteristic broad and strong band at 1100–900 cm^–1^ is indicative of the presence of an aluminosilicate skeleton.^38,39^ This is assigned to asymmetric Si–O and Al–O stretching vibrations of tetrahedral SiO_4_/AlO_4_.^40^ The presence of these bands was observed in all of the analyzed materials. The bands originating from EGCG exhibit partial overlap with the range of bands originating from the aluminosilicate structure. The peaks observed at 3480–3280 cm^–1^ are attributed to the O–H groups, while those at 1685 and 1615 cm^–1^ serve to confirm the presence of a C=O bond linking the trihydroxybenzoate group to the phenylchromane group. The bands at 1454 and 1345 cm^–1^ are attributed to the presence of a C–H group in the aromatic ring, while the O–C=O group is visible at 1225 cm^–1^. The presence of peaks at 1155 and 1092 cm^–1^ serves to confirm the presence of a hydroxyl group.^41^

The differences in band intensity in the spectra before and after the sorption process for samples modified with zinc zeolite demonstrate the efficacy of the process and the substantial amount of attached active substance. For samples with magnesium ions, the differences are much less pronounced. This result confirms the previous findings that a smaller amount of EGCG was attached to the surface of the magnesium zeolite. The absence of characteristic EGCG bands in the spectra of samples modified with calcium ions indicates that the added amount of active substance was minimal and that the sorption process was ineffective for this material.

The analysis conducted in ATR mode enabled the generation of maps illustrating the distribution of the active substance on the surface of the studied samples (Figure 6). Mapping was conducted for each sample over an area of 1000 × 1000 μm. The maps created before and after the sorption process were compared, and the color differences indicate the amount of the detected substance within a given scanning area. As illustrated by the scale accompanying the maps, the turquoise color indicates a minimal amount, while the purple color transitioning to pink signifies a considerable attachment of EGCG to the analyzed area. The maps obtained before the sorption process exhibited a uniform blue tone, suggesting the absence of the active substance on their surface. The greatest amount of EGCG was adsorbed by the surface modified with zinc ions, as evidenced by the intense purple-pink coloration across the entire map area. A significantly reduced amount of the active substance was retained on the magnesium zeolite-containing sample. In contrast, the entire area of the map derived from the sample with calcium zeolite is intensely turquoise, indicating that little to no EGCG was adsorbed on its surface.

**Figure 6:**
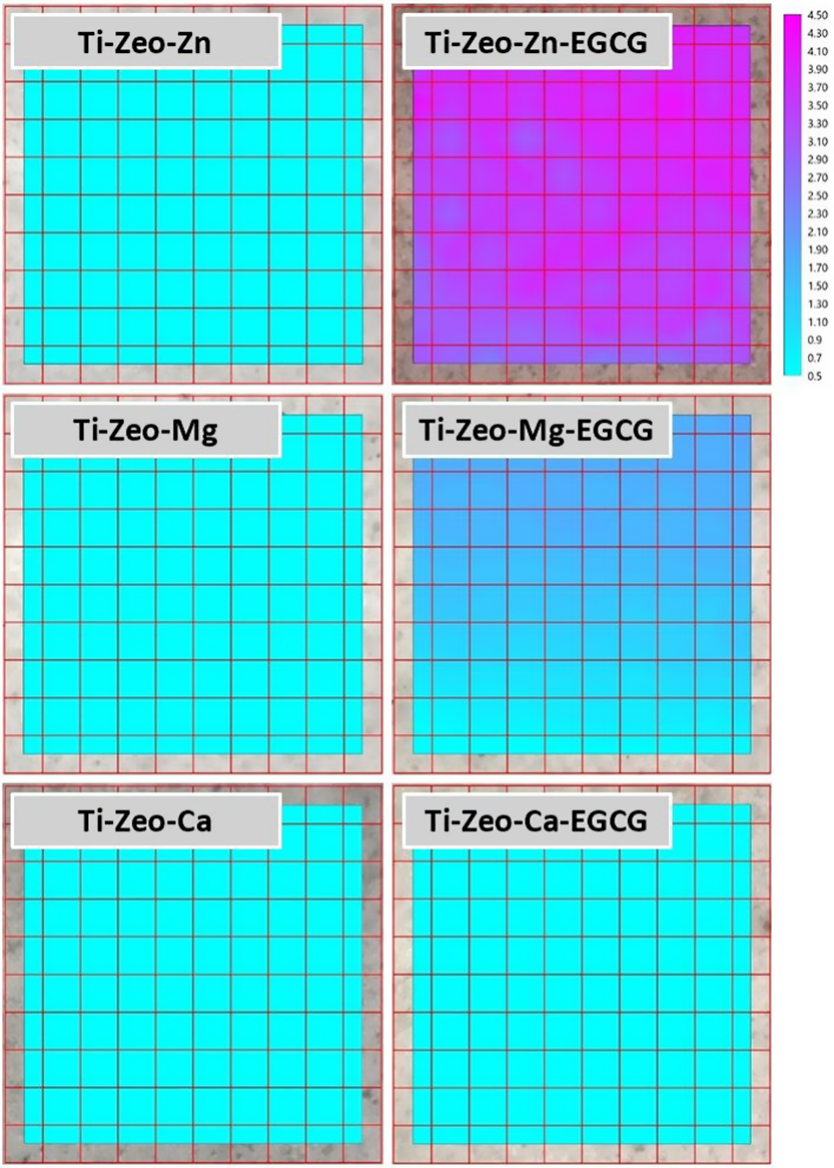
Distribution maps of EGCG on titanium alloy surfaces modified with zinc, magnesium, and calcium zeolite (Ti-Zeo-Zn, Ti-Zeo-Mg, and Ti-Zeo-Ca). The maps were generated based on the spectral band corresponding to EGCG in the 1450–1300 cm^–1^ range. The scale on the right indicates relative EGCG concentration on the surface, with higher values representing greater amounts of EGCG. The highest intensity is observed for Ti-Zeo-Zn-EGCG, a lower intensity for Ti-Zeo-Mg-EGCG, and the lowest intensity for Ti-Zeo-Ca-EGCG.

The observed differences in the amount of drug substance within a given scan area are minimal. This confirms that EGCG is distributed uniformly on both zinc and magnesium zeolites. This is particularly important for ensuring homogeneity and uniform coverage of the surface of the implantable material. A significant challenge is achieving uniform release of EGCG from the entire surface to prevent an excessively high dose from being released from specific regions. Such an outcome could result in a local overdose, which in turn could lead to irritation of surrounding tissues or inflammation. This could consequently prolong the regeneration period.

The exact amount of drug retained was determined using UV–vis analysis. The modified titanium samples were subjected to a five-day sorption process involving the active substance, EGCG. Each sample was placed in a solution with a concentration of 0.1 mg/mL, with a volume of 5 mL. The changes in solution concentration were analyzed using a UV–vis spectrophotometer at 1-, 2-, 3-, and 5-day intervals. The retained amounts of the active ingredient are illustrated in a bar graph (Figure 7).

**Figure 7:**
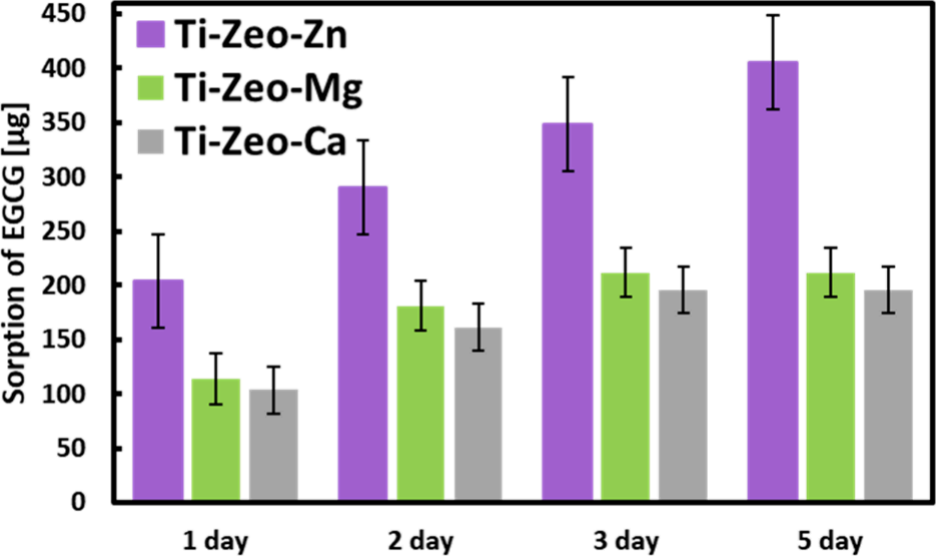
Amount of drugs retained on prepared materials (Ti-Zeo-Zn, Ti-Zeo-Mg, and Ti-Zeo-Ca). The error bars represent the standard deviation based on three measurements.

The analysis of Figure 7 indicates that the largest amount of EGCG was adsorbed by samples containing zinc zeolite. On the initial day, in excess of 200 μg of the therapeutic substance was retained on their surface. After 5 days, the amount had almost doubled. The sorption of the active substance on surfaces modified with zeolites containing magnesium and calcium ions proceeded at a similar level, with just over 100 μg retained after 1 day. In the case of these samples, no significant changes in the amount of adsorbed medicinal substance were observed between the third and fifth days of the process. Upon completion of the process, the retained amount of EGCG on the surfaces was approximately 200 μg.

The 2-fold increase in the amount of adsorbed therapeutic substance on surfaces containing zinc ions validates the conclusion drawn from previous analyses - detection of elements by EDS or the visualization of the active substance on maps from FT-IR microscopic analysis in ATR mode. The most effective carrier for the therapeutic substance EGCG is a titanium alloy modified with a zeolite containing zinc ions.

In order to examine the release of the previously adsorbed drug substance, the samples were placed in two different environments with distinct pH values. Therefore, SBF (simulated body fluid), with a high concentration of Na^+^ ions and acetate buffer, were used. Using two varying pH values will facilitate the examination of the EGCG release profile depending on the pH value present within the implant. The accumulation of cells responsible for bone resorption, osteoclasts, affects the pH of extracellular fluids, which under normal conditions has a value of approximately 7.4.^42^ The graphs below illustrate the release of EGCG at specified time intervals, with an estimation of the area released during the initial minutes and hours.

Based on the data presented in Figure 8, it can be concluded that the desorption profile of EGCG from surfaces modified with a specific zeolite in an SBF environment exhibits a correlation with the amount of adsorbed active substance. At specific time intervals, the amount of EGCG released from the surface containing zinc ions is markedly greater than the amount desorbed from the material modified with magnesium and calcium zeolite. During the first 3 h, EGCG desorption from the magnesium zeolite-coated titanium alloy surface occurred gradually, reaching a stable state after 1 day without an initial release of a significant amount of the active substance. Although sorption on the material containing calcium ions was at a similar level as on the material with magnesium ions, the amount released was almost five times smaller.

**Figure 8:**
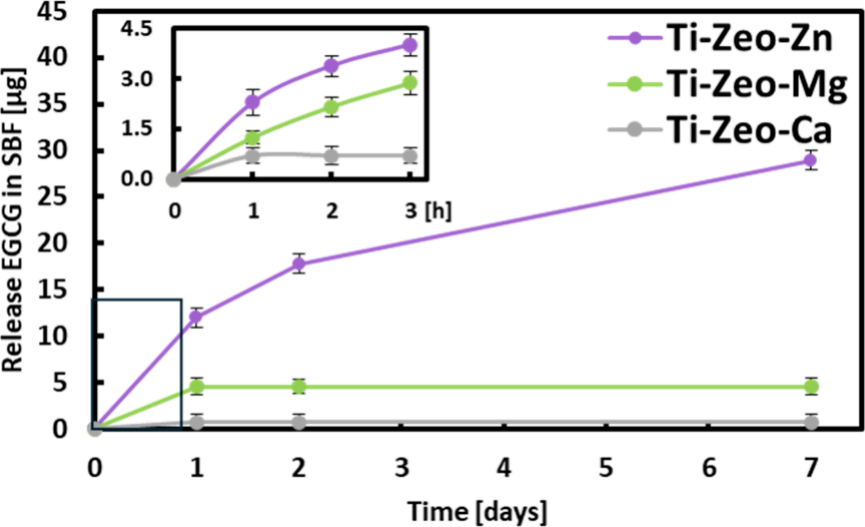
Amount of EGCG released from the materials (Ti-Zeo-Zn, Ti-Zeo-Mg, and Ti-Zeo-Ca) in simulated body fluid over a 7-day period. Error bars represent the standard deviation based on three independent measurements.

The analysis of Figure 9 for the amount of EGCG released in the acidic environment of acetate buffer indicates a more rapid desorption profile within the first day of the process. Following the initial release of a substantial amount of the active ingredient, the desorption profile tends to stabilize over the subsequent days for both the samples modified with zinc and magnesium zeolite. In the initial 75 min, it was observed that a greater amount of EGCG was released from the magnesium ion-modified surface in comparison to the zinc ion-containing surface. This phenomenon may be attributed to the enhanced complexation of the active substances by the zinc ions, resulting in a prolonged release process. In subsequent phases of the process, the amount of EGCG desorbed from the zinc ion-containing surface exceeds that released from the magnesium surface. This is attributed to the higher overall amount of the substance attached to the zinc surface. In contrast, the release profile of EGCG from the calcium zeolite-modified titanium alloy surface remains low throughout the test period, with a maximum of 5 μg released.

**Figure 9:**
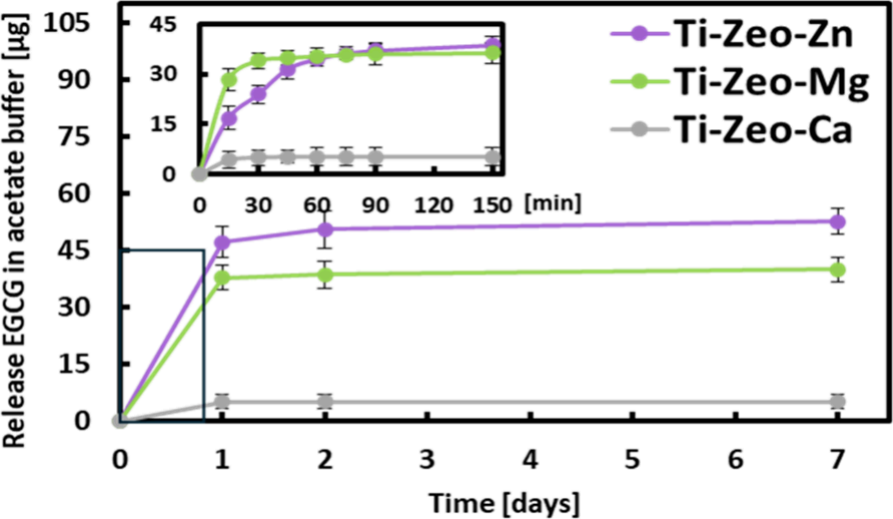
Release profile of EGCG from the materials (Ti-Zeo-Zn, Ti-Zeo-Mg, and Ti-Zeo-Ca) under the influence of acetate buffer over a period of 7 days. The graph shows the cumulative amount of EGCG released at different time points. Error bars indicate the standard deviation calculated from three independent measurements.

Comparing the release profiles of EGCG in two different environments (Figure 10) of different pH values, it was observed that when released in a neutral/lightly alkaline environment (SBF 7.4 pH), the active ingredient is released in small amounts over a longer period of time. This translates into a smoother desorption profile. In contrast, in the acidic environment of acetate buffer, the active ingredient is released in significant amounts on the first day. Subsequently, the profile exhibits stability, releasing consistent doses of the active ingredient at designated time intervals. It is noteworthy that, despite the stabilization of the release profile, EGCG will continue to be released by the subsequent ion exchange. The percentage of the active ingredient that was desorbed over the course of the study was calculated based on the amount that was adsorbed and released. The results are presented in Table 3.

**Figure 10:**
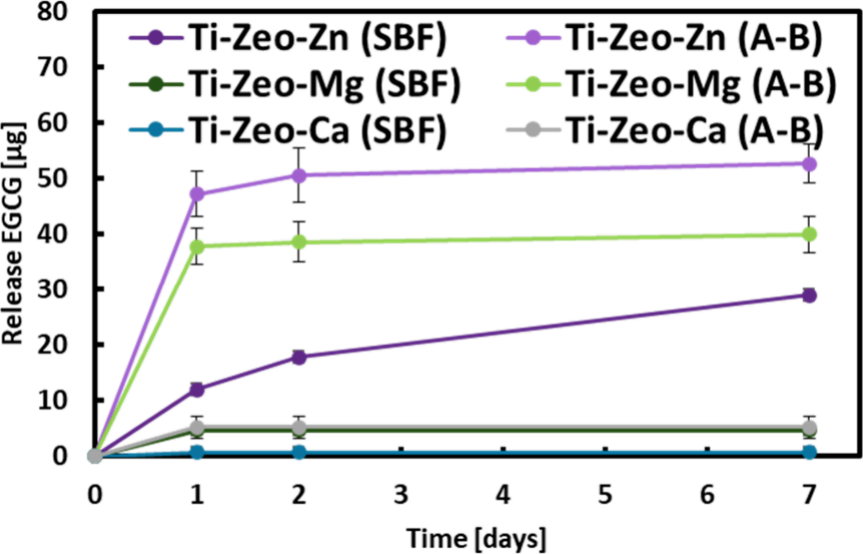
Comparison of EGCG desorption profiles from the materials (Ti-Zeo-Zn, Ti-Zeo-Mg, and Ti-Zeo-Ca) in simulated body fluid and acetate buffer over a 7-day period. Error bars represent the standard deviation based on three independent measurements.

**Table 3:** Percentage of Released Active Substances in a Given Environment

ion	SBF	acetate buffer
Zn^2+^	7.14%	13.00%
Mg^2+^	2.16%	18.87%
Ca^2+^	0.36%	2.65%

The long-term release of small doses of the therapeutic substance over an extended period of time enables the achievement of a sustained therapeutic effect. This approach minimizes the risk of toxicity and inflammatory reactions that may result from a local overdose. The controlled release of active substances via ion exchange represents an effective method of delivering drugs from the surface of implants, as it prevents reattachment of the therapeutic substance to the implant. The replacement of released active molecules with Na^+^ ions prevents their readsorption on the implant surface.

Studies show that EGCG is released in higher doses in an acidic environment at the initial stage of the process. In contrast, at pH 7.4, which corresponds to the value observed in body fluids, the release profile is relatively smoother. At specific time intervals, a smaller amount of the active substance is released. Furthermore, the analyses conducted indicate the potential for EGCG to inhibit the activity of a considerable number of osteoclasts. By lowering the pH value, these osteoclasts contribute to the ejection of a significant dose of EGCG, which has properties that inhibit its own activity.

The ideal solutions are a combination of EGCG action both under normal intrinsic conditions and under pathological conditions associated with osteoclast accumulation. The release of a significant dose of EGCG, when the pH value is lowered by the presence of osteoclasts, will enable the inhibition of their activity through its inhibitory action. This will help limit the progressive process of bone resorption. On the other hand, the controlled release of a small amount over an extended period in the body normal environment has been shown to have a beneficial effect on bone formation.^43–45^ Additionally, it is noteworthy that EGCG has high antibacterial activity, which can help to reduce inflammation and facilitate the regeneration of tissue around the implant.^46^

The previously described part of the study clearly confirms that the use of zeolite with divalent ions allows for controlled EGCG release, which is pH-dependent. It was also demonstrated that the type of ion plays a key role in the material’s performance. Based on previous analyses, the most promising material appears to be the one containing zinc ions. The magnesium-containing material shows lower, yet still promising, adsorption and release properties. However, the material with actual application potential will only be revealed in the next part of the study, which will present the cellular experiments. Since the calcium-ion-containing material did not release EGCG either in a neutral or an acidic environment, it was decided not to investigate its effect on cells.

### Biological Evaluation

#### Cytotoxicity Assessment

The results obtained from the MTT cytotoxicity test demonstrated that the Ti, Ti-Zeo-Mg and Ti-Zeo-Mg-EGCG variants were nontoxic with an excellent cell viability equal to 104 and 103%, respectively. Whereas the Ti-Zeo-Zn and Ti-Zeo-Zn-EGCG variants resulted in strong cytotoxicity on hFOB 1.19 cells and complete cell lysis (Figure 11). This indicates that Zn modification was unfavorable for human osteoblasts and should not be considered in further work on the implant. It should be noted that although Zn^2+^ ions are known to promote bone regeneration process,^47^ their high concentration is lethal to the eukaryotic cells.^48,49^ Thus, a great number of Zn-based biomaterials, that are described in the available literature, show cytotoxic effects. For instance, Tong et al. revealed that Zn-based alloys are characterized by strong cytotoxicity toward mouse MC3T3-E1 preosteoblasts.^49^ Similarly, Su et al. demonstrated that 100% extracts of Zn disc, ZnP-coated Zn disc and collagen-coated Zn disc critically decreased viability of MC3T3-E1 cells by >90%.^50^

**Figure 11:**
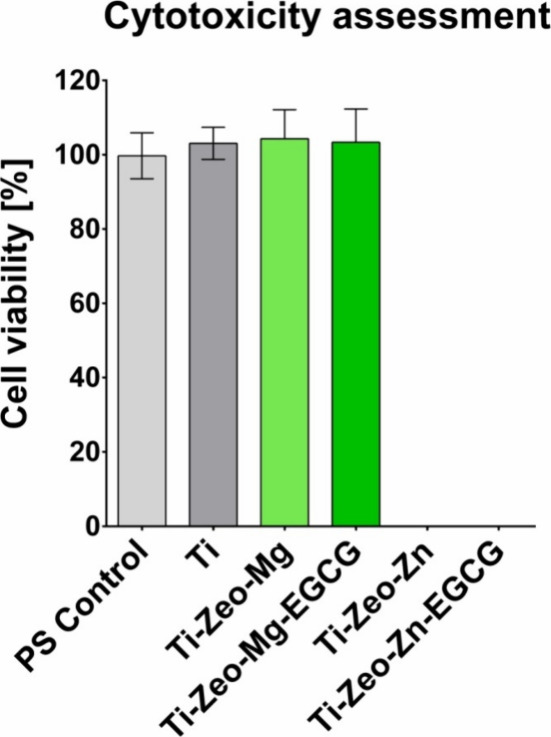
Cytotoxicity assessment according to ISO 10993-5 using the hFOB 1.19 cell line and MTT tests performed after 24 h of incubation with biomaterials extracts.

Live/dead staining of hFOB 1.19 cells cultured directly on the materials surfaces showed that only unmodified Ti samples supported osteoblasts adhesion and flattening. The test confirmed high cytotoxicity of Ti-Zeo-Zn and Ti-Zeo-Zn-EGCG variants as no viable cells were detected on their surfaces, which was not surprising considering MTT results. Moreover, only single dead red fluorescent cells were observed on these samples, proving complete cell lysis and nuclei degradation after seeding osteoblasts on Ti-Zeo-Zn and Ti-Zeo-Zn-EGCG samples. The lack of a strong red signal emitted by dead cells was also due to unfavorable cell adhesion to biomaterial surface, i.e. very few cells managed to attach to the sample surface, and therefore only single dead cells were detected. The Ti-Zeo-Mg and Ti-Zeo-Mg-EGCG biomaterials were covered by viable green fluorescent cells and nontoxic (no red signal detected), confirming quantitative MTT results. However, surface of the samples did not support cell adhesion since there were noticeably fewer hFOB 1.19 osteoblasts than on the Ti sample and the osteoblasts showed spherical morphology typical of unattached cells (Figure 12). This feature is highly favorable in the case of temporary implants, which require surgical removal after completed healing process.^51^ If such an implant supports the adhesion of bone-forming cells to its surface, its removal from the bone after the convalescence period would be associated with unnecessary additional damage to the bone tissue. In ideal conditions, such an implant should be removable with the least traumatic injury to healed bone.

**Figure 12:**
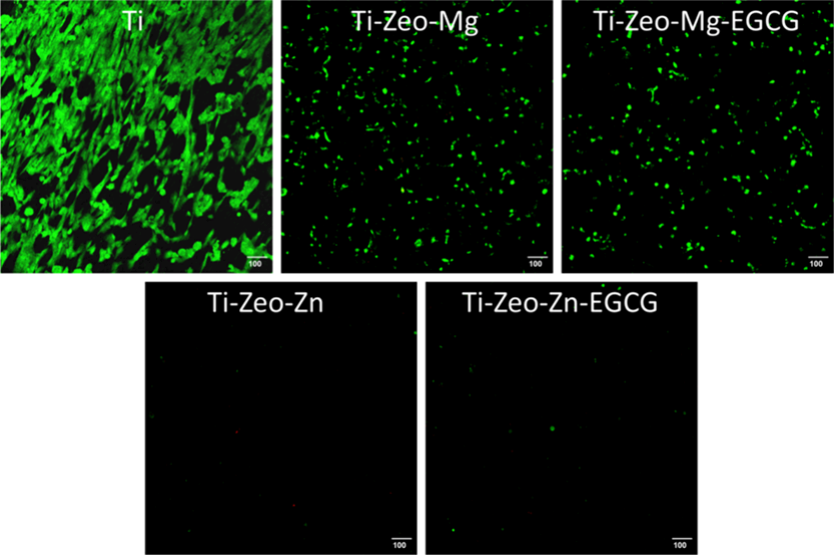
Live/dead staining of hFOB 1.19 cells cultured on the material surfaces. Green fluorescence: viable cells, red fluorescence: dead cells.

#### Cell Proliferation Assessment

According to results obtained from cytotoxicity test, cell proliferation assay was conducted using only nontoxic materials: Ti, Ti-Zeo-Mg and Ti-Zeo-Mg-EGCG. An increase in cell number over time was observed, however proliferation potential was significantly higher for control cells cultured on titanium (Ti) and polystyrene (control PS) surface (Figure 13).

**Figure 13:**
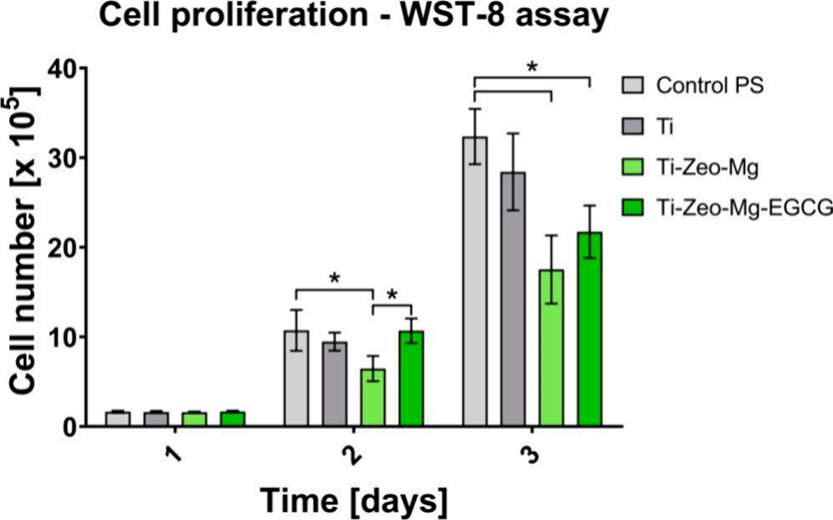
Cell proliferation assessment. * Means statistically significant differences between indicated groups, *p* < 0.05.

## Conclusions

This study provides a comprehensive evaluation of titanium-based implants functionalized with zeolite coatings as advanced drug delivery platforms for bone regeneration. The integration of divalent ions-zinc, magnesium, and calcium-into the zeolite matrix was shown to significantly influence both EGCG adsorption and controlled release, highlighting the critical role of ion selection in tailoring implant functionality. While zinc-zeolite surfaces demonstrated the highest EGCG loading, cytotoxicity studies revealed their cytotoxicity toward human osteoblasts, underscoring the importance of balancing therapeutic potential with cellular safety. Magnesium-zeolite coatings offered a favorable compromise, enabling pH-sensitive, sustained EGCG release: low doses in physiological conditions promote osteogenesis, whereas higher doses in acidic microenvironments, created by osteoclast activity, inhibit excessive bone resorption. Surface topography analyses confirmed that coating complexity enhances the biologically active area, optimizing drug delivery without promoting undesirable cell adhesion, which is crucial for temporary internal fixation. Together, material characterization, release kinetics, and biological evaluation demonstrate the feasibility of designing safe, removable, and therapeutically active implants. This work establishes a framework for the development of multifunctional implants capable of providing mechanical support while locally modulating bone remodeling and supporting recovery in osteoporotic conditions. The findings advance the concept of combining structural biomaterials with targeted pharmacological activity, offering a promising avenue for next-generation orthopedic devices.

## Data Availability

Data supporting the findings of this study are available in the open RepOD repository with the identifier 10.18150/TTOAOE.
